# Analysis of neuronal ensemble activity reveals the pitfalls and shortcomings of rotation dynamics

**DOI:** 10.1038/s41598-019-54760-4

**Published:** 2019-12-12

**Authors:** Mikhail A. Lebedev, Alexei Ossadtchi, Nil Adell Mill, Núria Armengol Urpí, Maria R. Cervera, Miguel A. L. Nicolelis

**Affiliations:** 1 Duke Center for Neuroengineering, Duke University, Durham, NC USA; 2 Center for Bioelectric Interfaces of the Institute for Cognitive Neuroscience of the National Research University Higher School of Economics, Moscow, Russia; 3 Institute of Neuroinformatics, University of Zurich and ETH Zurich, Zurich, Switzerland; 4 D-MAVT ETH Zurich, Zurich, Switzerland; 5 Department of Neurobiology, Duke University Medical Center, Durham, NC USA; 6 Department of Neurology, Duke University, Durham, NC USA; 7 Department of Neurosurgery, Duke University, Durham, NC USA; 8 Department of Psychology and Neuroscience, Duke University, Durham, NC USA; 9 Edmond and Lily Safra International Institute of Neurosciences of Natal, Natal, Brazil

**Keywords:** Neuroscience, Sensorimotor processing

## Abstract

Back in 2012, Churchland and his colleagues proposed that “rotational dynamics”, uncovered through linear transformations of multidimensional neuronal data, represent a fundamental type of neuronal population processing in a variety of organisms, from the isolated leech central nervous system to the primate motor cortex. Here, we evaluated this claim using Churchland’s own data and simple simulations of neuronal responses. We observed that rotational patterns occurred in neuronal populations when (1) there was a temporal sequence in peak firing rates exhibited by individual neurons, and (2) this sequence remained consistent across different experimental conditions. Provided that such a temporal order of peak firing rates existed, rotational patterns could be easily obtained using a rather arbitrary computer simulation of neural activity; modeling of any realistic properties of motor cortical responses was not needed. Additionally, arbitrary traces, such as Lissajous curves, could be easily obtained from Churchland’s data with multiple linear regression. While these observations suggest that temporal sequences of neuronal responses could be visualized as rotations with various methods, we express doubt about Churchland *et al*.’s bold assessment that such rotations are related to “an unexpected yet surprisingly simple structure in the population response”, which “explains many of the confusing features of individual neural responses”. Instead, we argue that their approach provides little, if any, insight on the underlying neuronal mechanisms employed by neuronal ensembles to encode motor behaviors in any species.

## Introduction

It is well known that individual neurons in cortical motor areas transiently modulate their firing rates following a stimulus that triggers the production of a voluntary movement^1^. These neuronal modulations have been shown to represent various motor parameters, for example movement direction^2^, although the specifics of these representations are still a matter of debate^3–5^. With the development of multichannel recordings^6,7^, it has become possible to study modulations recorded in multiple cortical neurons simultaneously. This methodological advance led to many studies attempting to uncover how neuronal populations process information^8–11^.

**Figure 1:**
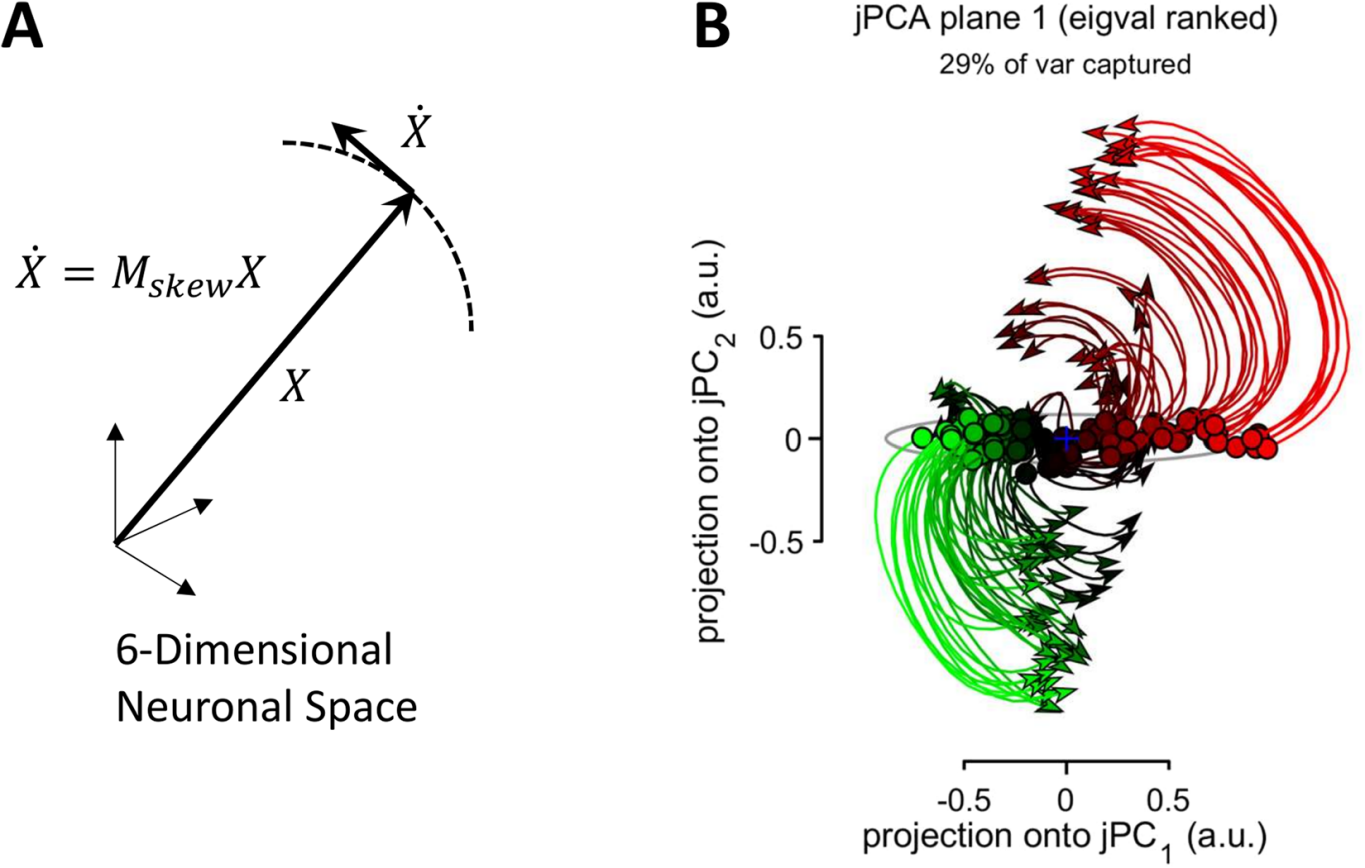
Rotation in a multidimensional neuronal space. (**A**) Schematics of “rotational dynamics”, where there is an angle between the neuronal vector, *X*, and its time derivative, $\dot{X}$, meaning that *X* is turning. In jPCA proposed by Churchland *et al*. (2012), is *X* is formed by the first six PCs of the population activity. (**B**) Application of jPCA to neuronal data. Individual curves correspond to experimental conditions, where a monkey performed armed reaching with different trajectories. The color of the curves (red, green, black) corresponds to different levels of premovement activity.

Among the studies on motor and premotor cortical neuronal populations, one paper by Churchland and his colleagues^12^ became especially popular. In their work, Churchland *et al*. claimed to have discovered a unique property of cortical population activity that they called “rotational dynamics”. Their analysis is based on the idea that motor cortical activity could be modeled as a dynamical system (the idea that is currently being radically expanded by new machine learning techniques^13^):

1 $$\dot{X}={M}_{skew}X$$

where *X* is a multidimensional vector representing neuronal population activity, $\dot{X}$ is its time derivative and *M*_*skew*_ is the transform matrix. *M*_*skew*_ has imaginary eigenvalues, meaning that $\dot{X}$ is orthogonal to *X*^14^, which corresponds to *X* rotating around the center of rotation at the center of coordinates (Fig. 1A). (Churchland *et al*. did not explain why rotations around a fixed center are of particular interest as opposed, for example, to curved trajectories with a varying center of rotation). In the analysis of Churchland *et al*., vector *X* is produced from the activity of neuronal populations by the application of principal component analysis (PCA). The first six principal components (PCs) are selected to avoid overfitting that could take place with a larger number of dimensions. Hence, *X* is six-dimensional. Next, a method called jPCA is applied to compute *M*_*skew*_ and the corresponding eigenvectors. Churchland *et al*. projected PC data to the plane defined by the first two most prominent rotational components generated with jPCA and obtained the figures, where population responses rotated in the same direction for different experimental conditions (Fig. 1B).

According to the interpretation of Churchland *et al*., “rotational dynamics” are a fundamental feature of population activity in the motor and premotor cortical areas (but not the supplementary motor area^15^) and a proof that motor cortical populations act as a dynamical system rather than representing various motor parameters by their firing. They called the rotational effect an “orderly rotational structure, across conditions, at the population level”, a “brief but strong oscillatory component, something quite unexpected for a non-periodic behavior”, and “not trivial” observations that are “largely expected for a neural dynamical system that generates rhythmic output”.

While this proposal has merits, we found the results of Churchland *et al*. difficult to comprehend because of the lack of clarity regarding the concrete neuronal patterns contributing to the rotations revealed by jPCA. For example, they suggested that “motor cortex responses reflect the evolution of a neural dynamical system, starting at an initial state set by preparatory activity […]. If the rotations of the neural state […] reflect straightforward dynamics, then similar rotations should be seen for all conditions. In particular, the neural state should rotate in the same direction for all conditions, even when reaches are in opposition” – a high-level description that lacks concrete details regarding activity patterns of the neurons entered in the analysis. For instance, it is unclear what “straightforward dynamics” are and why they impose the same rotational patterns on all conditions. The major obstacle to understanding the ideas expressed by Churchland and his colleagues is the absence of a clear explanation of how individual neurons and/or their populations form the rotating patterns revealed by jPCA.

To eliminate this gap in understanding, we reanalyzed some of the data that Churchland *et al*. made available online. We utilized perievent time histograms (PETHs), a basic method for displaying neuronal data, to find the underlying cause for the “rotational dynamics”. Next, we ran simple simulations to verify our findings. Based on our results, we found several issues with the analyses and interpretations by Churchland *et al*. Even though a certain temporal order in which different cortical neurons are activated during a behavioral task could be described as “rotational dynamics”, we are not convinced that this observation alone could significantly “help transcend the controversy over what single neurons in motor cortex code or represent”, as stated by Churchland and his colleagues.

## Results

Rotation in a multidimensional neuronal space could be thought of as a process where individual neurons are activated in a certain order, which results in the neuronal vector *X* changing orientation (Fig. 1A). Such a pattern can be also described as a phase shift between the responses of different neurons. Consider the simplest case of a population that consists of two neurons where the activity of the first neuron is a sine function of time and activity of the second neuron is a cosine. Since the phase shift between the responses of these neurons is 90 degrees, a two-dimensional plot with the firing rates of these neurons on the axes produces circular or elliptical trajectories. This type of trajectory is observed for all conditions if the phase shift between the neurons persists.

**Figure 2:**
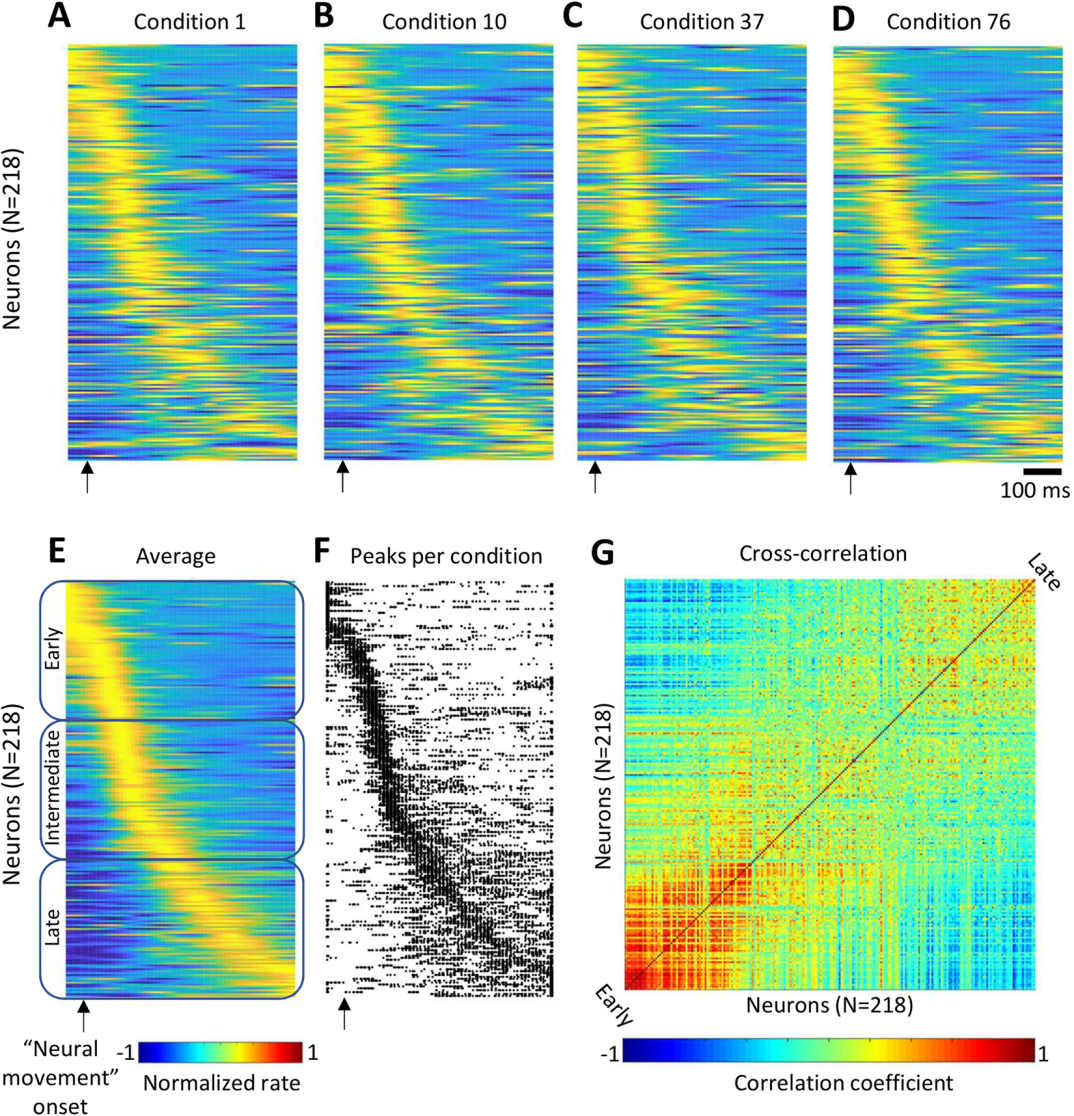
Time spread in peak firing rates of different neurons in the data provided by Churchland *et al*. (2012). (**A–D**) Color-coded population PETHs for four representative conditions. Horizontal lines represent PETHs for individual neurons. (**E**) PETHs averaged across conditions. From top to bottom: neurons are ordered by the time of peak firing rate, from the earliest activated neurons to the latest. The same order is used in (**A–D**,**F**,**G**). The neuronal population was dived into three subpopulations: early (neurons 1–73), intermediate (74–146), and late (147–218) responses. (**F)** A scatterplot showing the times of peak firing rates for different neurons and conditions. The times of peak  occurrences are represented by dots. (**G**) Pairwise correlation between the activity patterns of different neurons.

Following this logic, we hypothesized that the data of Churchland *et al*. contained phase shifts between the responses of different neurons, which remained consistent across conditions. To test this hypothesis, we analyzed their data using the traditional PETH method. The dataset included PETHs of 218 neurons calculated for 108 experimental conditions (i.e., one smoothed PETH per condition; single-trial data were unavailable). Each condition corresponded to a monkey performing a reaching movement with a straight or convoluted trajectory. We simply stacked these PETHs to produce population color plots for different conditions (Fig. 2A–D). Additionally, we averaged the PETHs across all conditions to obtain average responses for each neuron (Fig. 2E). For the average PETHs, we calculated peak values and reordered the neurons according to the value of the time when each neuron reached its peak firing rate. In the color plot showing the average PETHs (Fig. 2E), PETHs of the neurons activated early are plotted at the top and PETHs of the neurons activated late are plotted at the bottom, which results in a clear display of an activity wave running across the neurons in the population. This exact reordering was applied to the PETHs for individual conditions (Fig. 2A–D). In the PETHs for individual conditions, the temporal order of responses persisted with some jitter (e.g., compare panels A, B, C and D in Fig. 2). The same sequence of responses is also clear in the scatterplot that displays the time of peak response for different conditions and different neurons (Fig. 2F). Additionally, pairwise correlations were strong for the neurons with similar occurrences of response and weak (or negative) for the neurons with dissimilar occurrences (Fig. 2G). Thus, the PETH analysis showed that in Churchland’s data neurons responded in a certain temporal order, and this order persisted to an extent when the monkey altered the way it performed a reaching movement.

**Figure 3:**
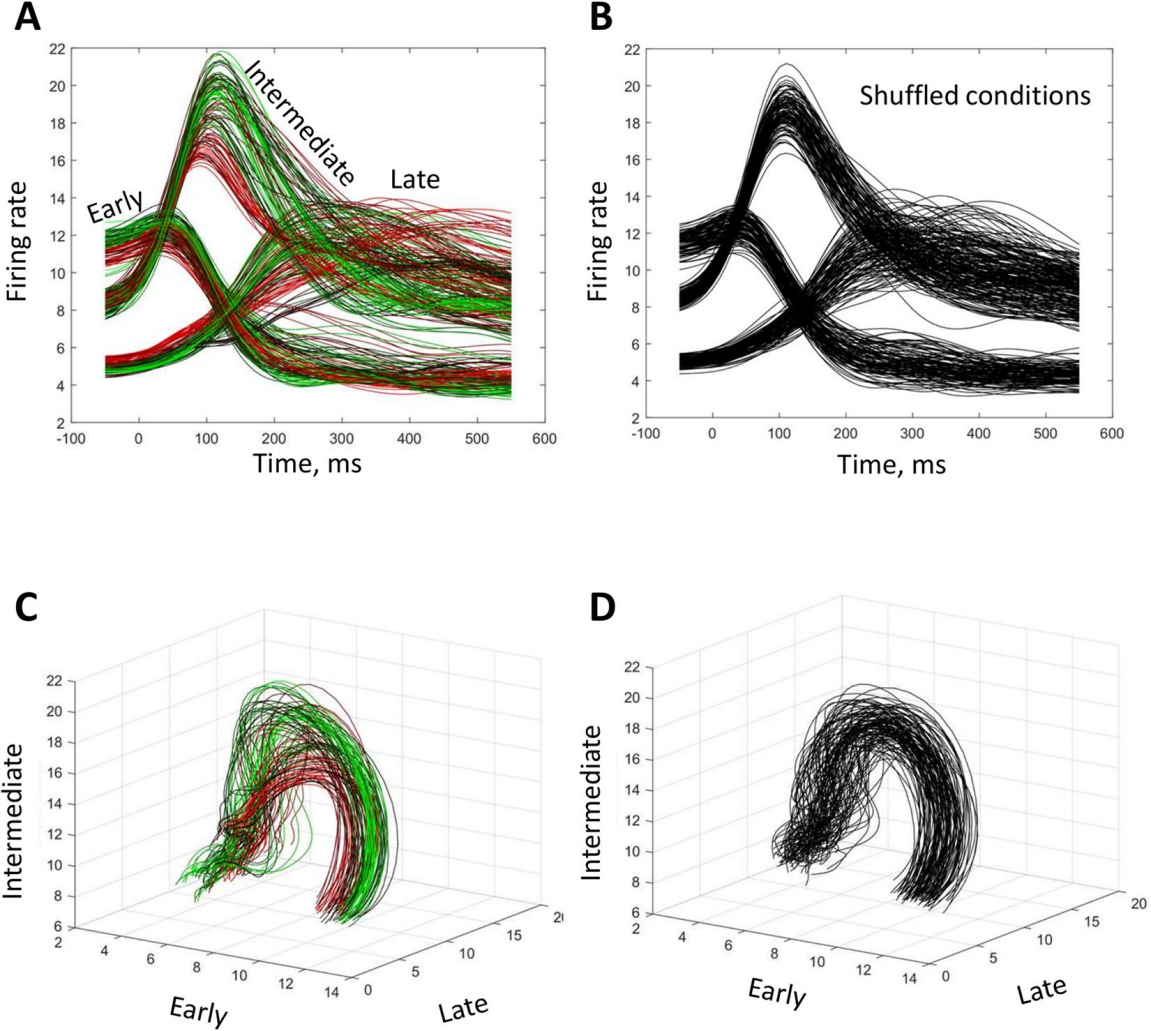
“Rotational dynamics” revealed by splitting neuronal population into the early, intermediate and late activated subpopulations. (**A**) Average PETHs for the three subpopulations, for different experimental conditions. The composition of the subpopulations is shown in Fig. 2E. Color coding of the traces is the same as in Fig. 1B. (**B**) Average PETHs for the shuffled-conditions data. (**C,D**) “Rotational dynamics” shown as three-dimensional plots with the axes representing average PETHs for different subpopulations. Original (**C**) and shuffled (**D**) datasets are shown.

To assess how the activation order of different neurons could contribute to a population rotational pattern, we split the entire neuronal population into three subpopulations: neurons with early peaks (ranks 1–73), intermediate peaks (74–146), and late peaks (147–218) (Fig. 2E). Next, we calculated average PETHs for each subpopulation and for each experimental condition (Fig. 3A). As expected, this plot revealed three groups of PETHs (early-peak, intermediate-peak and late-peak) whose shapes did not change substantially across conditions. Plotting these PETHs in a three-dimensional space (where dimensions corresponded to the subpopulations) yielded a family of curved trace (Fig. 3C) that resembled the circles obtained with jPCA (Fig. 1B). As an additional control, we randomly shuffled conditions for each neuron and plotted the curves for the shuffled data (Fig. 3B,D). Both the average PETHs for the subpopulations (Fig. 3B) and the three-dimensional plot (Fig. 3D) were little affected by the shuffling procedure, which confirmed that the activation order of the neurons was roughly the same for different conditions.

**Figure 4:**
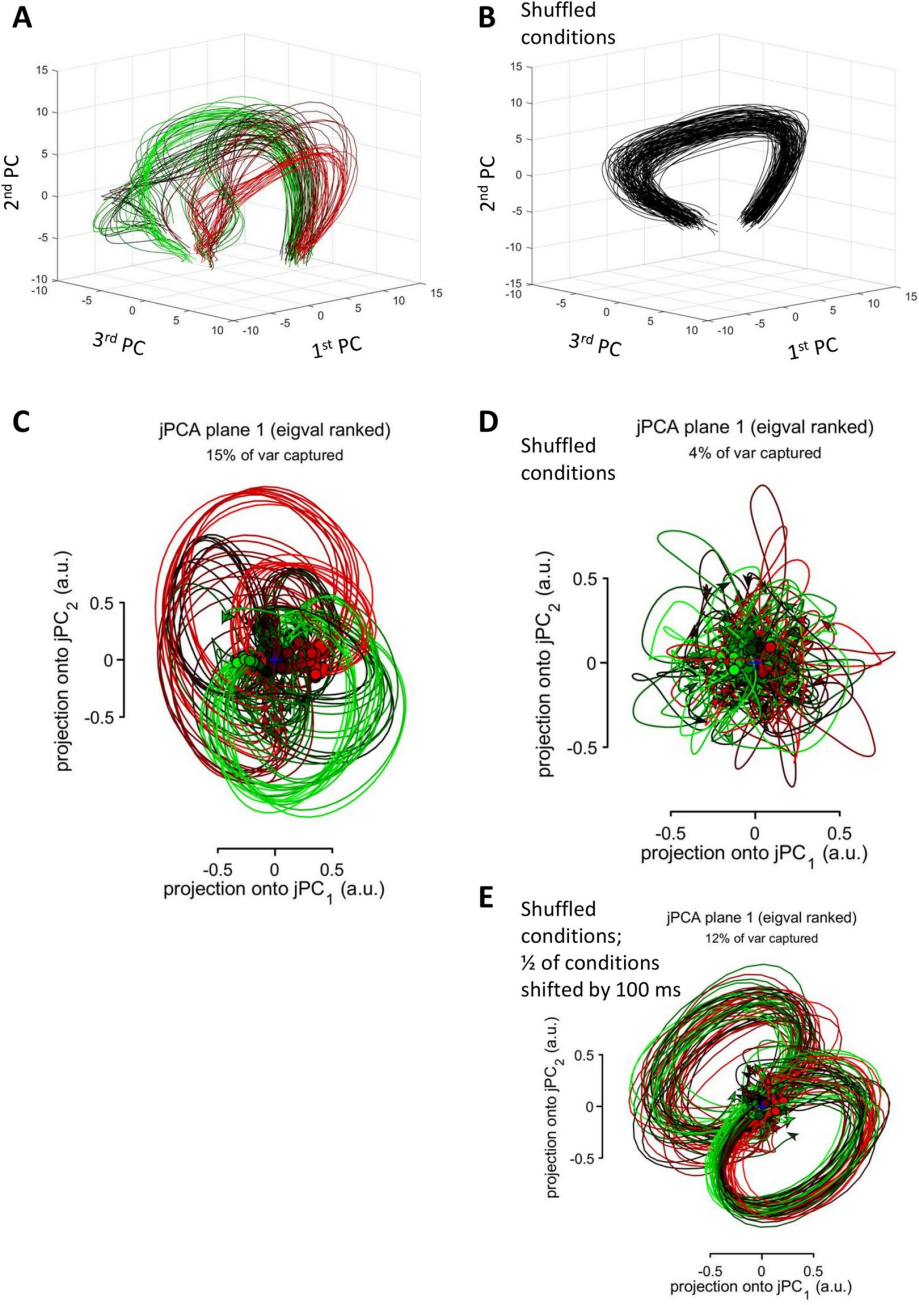
Rotations of principal components. (**A**) The first three PCs plotted as a three-dimensional plot. Color conventions as in Fig. 1B. (**B**) PCs for the data with shuffled conditions. (**C**) jPCA results for the data in (**A**). (**D**) jPCA results for the data in (**B**). (**E**) jPCA results after the shuffled data (**B**) was shifted forward by 100 ms for one-half of the conditions.

To further clarify the origin of the rotational patterns, we calculated the initial three PCs for the data of Churchland *et al*. and plotted them as a three-dimensional plot (Fig. 4A). The PC traces were clearly curved. Additionally, distinct clusters of conditions were visible in the plots, each of them containing several traces that had similar shapes. The clusters started from approximately the same point but separated toward the end of the trial. Despite the differences between the clusters, they rotated in approximately the same fashion (e.g., in the plane defined by the first and second PCs). Thus, “rotational dynamics” were clearly visible even before the application of jPCA. As to JPCA, it also yielded several clusters of circles (Fig. 4C).

Rotations of PC traces remained even after experimental conditions were randomly shuffled for each neuron (Fig. 4B), which indicated that condition-specific correlation between the responses of different neurons was not crucial for a rotational pattern to occur. While the PC traces for the shuffled data were clearly curved, they were not clustered any more after the shuffling procedure. Unlike PCA results, jPCA failed to detect these obvious curvatures and returned noisy traces (Fig. 4D). This result suggested that, after the shuffling procedure made the population responses very similar for different conditions, the data became ill-conditioned^16^ for jPCA. We, however, found a simple solution to this problem. We introduced variability to the entries for different conditions: for half of the conditions (conditions 55–108), the PETHs of all neurons were shifted forward by 100 ms, and the initial 100 ms of the PETHs were assigned constant values that were equal to the first point of each original PETH. Such activity pattern would have occurred if the monkey delayed movement initiation by 100 ms. Importantly, this manipulation did not alter the temporal sequence of neuronal population. Following the introduction of this shift, jPCA returned circles (Fig. 4E).

As a side note, jPCA with default settings encounters the same problem for a simple simulation where responses of a half of the neurons are a sine function and responses of the other half are a cosine. Although this is an obvious case of the presence of “rotational dynamics”, jPCA fails to generate circles. However, the circles appear after the population responses are shifted in time by a random amount for different conditions. As explained below, the problem of ill-conditioning occurs because jPCA incorporates by default a data preprocessing step where, for each neuron, an across-condition average PETH is subtracted from all PETHs for that neuron.

**Figure 5:**
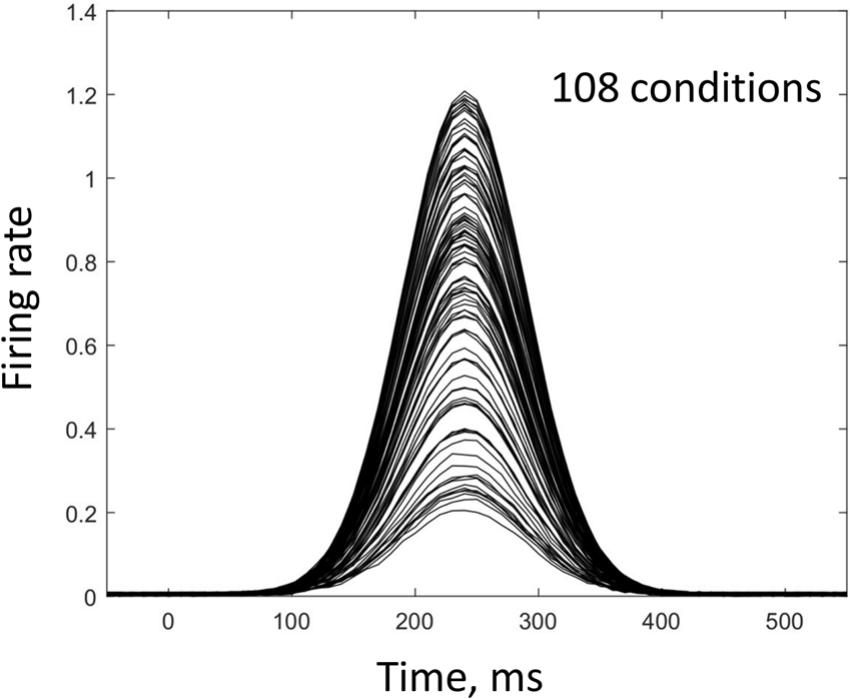
Simulated responses for an individual neuron. Neuronal responses were simulated using a Gaussian function. Response amplitude was randomly drawn for each condition from a uniform distribution (in the interval 0.2–1.2).

Having established that neurons were activated in consistent temporal order in Churchland’s data, we examined this effect further using a simple simulation of population activity. This simulation did not incorporate any features specific for motor cortical responses. For example, simulated neurons were not directionally tuned and instead each of them exhibited random amplitude of response for different conditions. Because of this randomness, response amplitude was not correlated in any pair of neurons. The only nonrandom pattern that we simulated was the presence of a temporal sequence in the responses in different neurons. The shape of simulated PETHs was a Gaussian function with an amplitude drawn from a uniform distribution (Fig. 5). We simulated 218 neurons and 108 conditions to match Churchland’s data.

Recently, Michaels, *et al*.^17^ used a somewhat similar simulation of motor cortical neurons with a temporal sequence of responses that was consistent across conditions. Like our simulation, they observed an occurrence of “rotational dynamics” under these conditions. Their simulation was, however, more sophisticated than ours as they simulated directionally tuned motor cortical neurons and attempted to compare a representational model of population activity with a dynamical-system model. To prove that jPCA reveals “complex aspects” of motor cortical activity, they designed sophisticated methods for data permutations. Yet, they did not consider the possibility that some of these permutations made their simulated data ill conditioned, which resulted in jPCA failing to return circular trajectories. In our simulation, resemblance to motor cortical activity was minimal. We also applied a simple correction to cope with the ill conditioned data. Moreover, our overall conclusion is the opposite to the opinion of Michaels *et al*. who highly valued jPCA approach and used it as the gold standard for assessing the results of their simulation. In addition to the study of Michaels *et al*., other mentions can be found in neurophysiological literature of the relationship between neuronal sequences and rotations found in PC planes. Thus, Hall *et al*.^18^ noted that “rotation in the PC plane does not require underlying oscillatory sources that are orthogonal, because any consistent phase difference, or a traveling wave appearing with a different phase on each electrode, could equally be decomposed into orthogonal components”, and Xu *et al*.^19^ reasoned that “state-space rotation implies neurons are consistently active at different phases of a cycle”.

**Figure 6:**
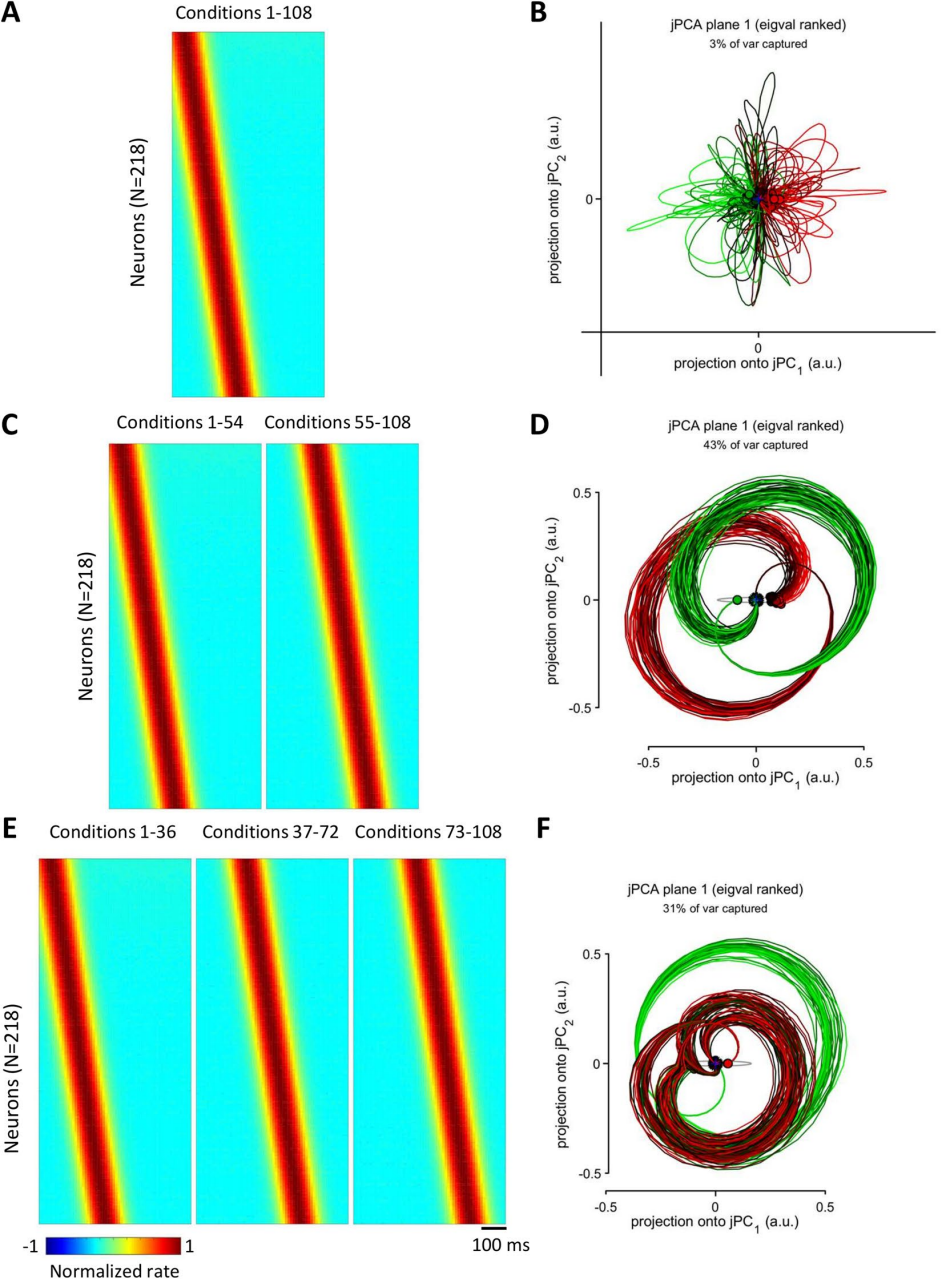
Simulated population responses with a time spread of peak firing rates. (**A**) PETHs for a simulated wave of population activity, where response of neuron *i* occurs 1 ms later than response of neuron *i-1*. (**B**) jPCA results for the data in (**A**). (**C**) The same population wave as in A with an early onset for conditions1–54 and late onset for conditions 55–108. (**D**) jPCA results for the data in (**C**). (**E**) The same population wave as in (**A**,**C**) with three different onsets for conditions 1–36, 37–72 and 73–108. (**F**) jPCA results for the data in (**E**).

We started with a simulation, where the responses of different neurons were shifted in time: neuron *i* responded 1 ms later than neuron *i-1* (Fig. 6A). This simulation produced a wave of activity running through the neuronal population. Here, the population responses were very similar for different conditions as in the results for shuffled data presented above. Apparently, in this case the data was ill-conditioned for jPCA, and this is why this analysis returned noise (Fig. 6B) even though the input data contained a consistent temporal sequence of neuronal responses. To make neuronal responses more variable across conditions and thereby remove the ill conditioning, we simulated two groups of conditions: for conditions 1–54, the first neuron exhibited peak activity at time *t* = 50 ms, and for conditions 55–108 the time of its peak activity was *t* = 200 ms (Fig. 6C). The structure of the activity wave (i.e. the rule that neuron *i* responded 1 ms later than neuron *i-1*) was the same for both groups of trials. This slight alteration of the data, which did not change the structure of the population response, was sufficient for jPCA to start generating circles (Fig. 6D). We also simulated three groups of conditions, where the first neuron’s peak rate occurred at 50, 150 and 200 ms for the first, second and third groups, respectively (Fig. 6E). The structure of the population wave was the same for all three groups. In this case, again, jPCA returned circles (Fig. 6F).

**Figure 7:**
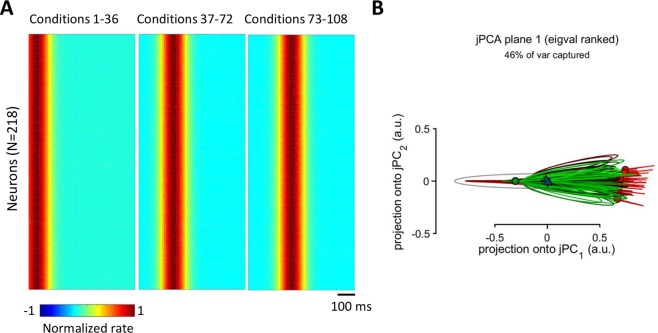
Simulated population responses without a time spread of peak firing rates. (**A**) PETHs with different response onsets for conditions 1–36, 37–72 and 73–108. For each group of conditions, peak responses of different neurons were not spread in time. (**B**) jPCA results for the data in (**A**).

To verify that a temporal sequence of activation was necessary for the rotation pattern to occur for our simple model of neuronal responses, we simulated a neuronal population where all neurons responded simultaneously (Fig. 7). The shapes of neuronal responses were the same as in the previous simulation (Fig. 5). In this case, jPCA failed to generate circles (Fig. 7B) even when three groups of conditions were simulated with shifted activity onsets (Fig. 7A).

**Figure 8:**
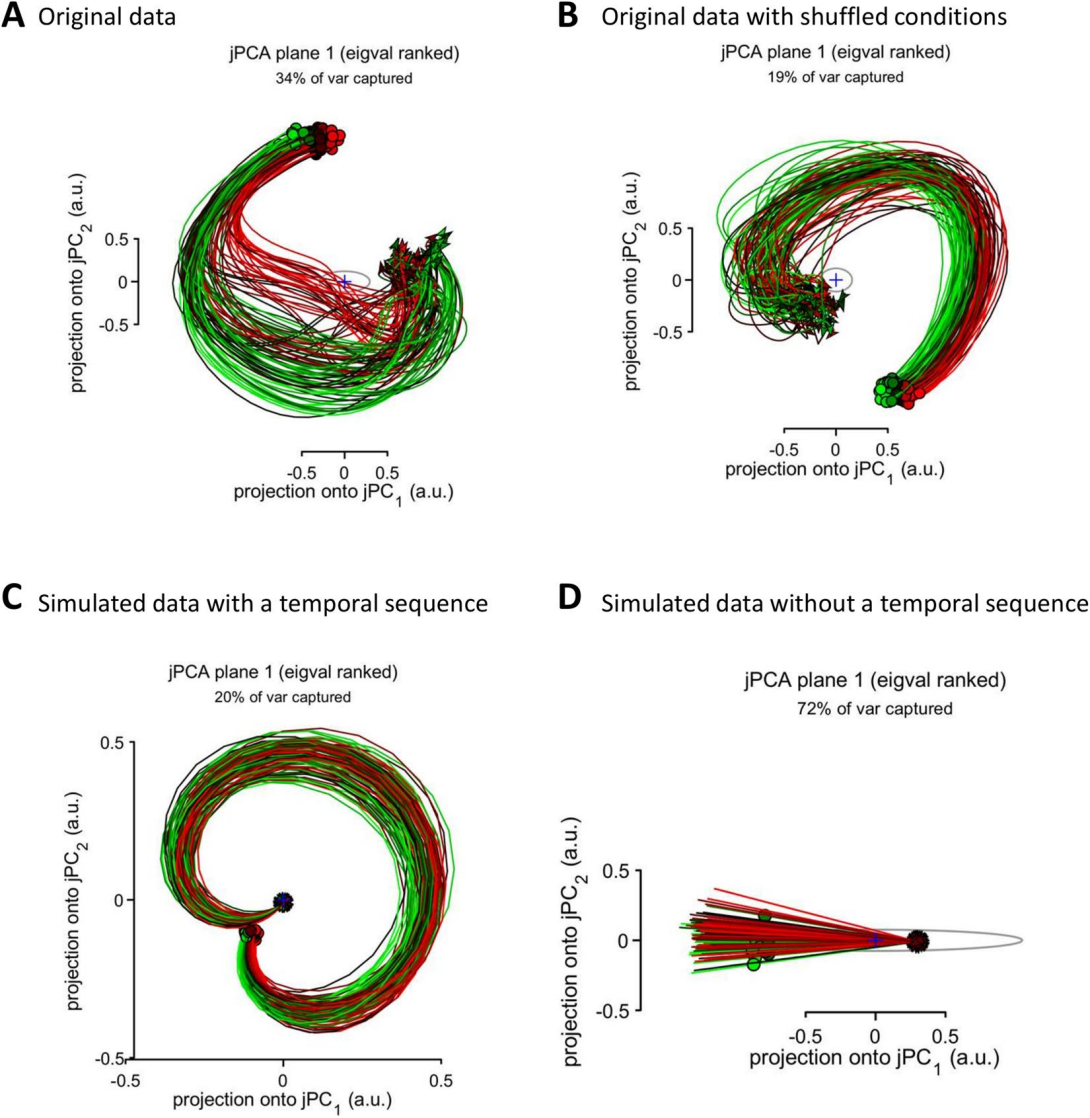
jPCA results without subtracting across-condition average responses. (**A**) Original data of Churchland *et al*., the same data as in Fig. 4C. (**B**) Original data after across-condition shuffling, the same data as in Fig. 4D. (**C**) Simulated data with a temporal sequence of responses, the same data as in Fig. 5A,B. (**D**) Simulated data without a temporal sequence of responses, the same data as in Fig. 7.

In addition to running jPCA with the default setting, where an average across-condition response was subtracted from each PETH, we ran jPCA without this setting (Fig. 8). In this case, the problem of ill conditioning did not occur, and jPCA returned circles in all cases with the exception of the simulation, where there was no temporal sequence of neuronal responses (Fig. 8D). Overall, probing our simulated data with different versions of Churchland’s jPCA supported the hypothesis that a temporal sequence of neuronal responses results in a population rotation pattern, with a caveat that the problem of data ill-conditioning had to be handled when jPCA with the default settings was applied.

**Figure 9:**
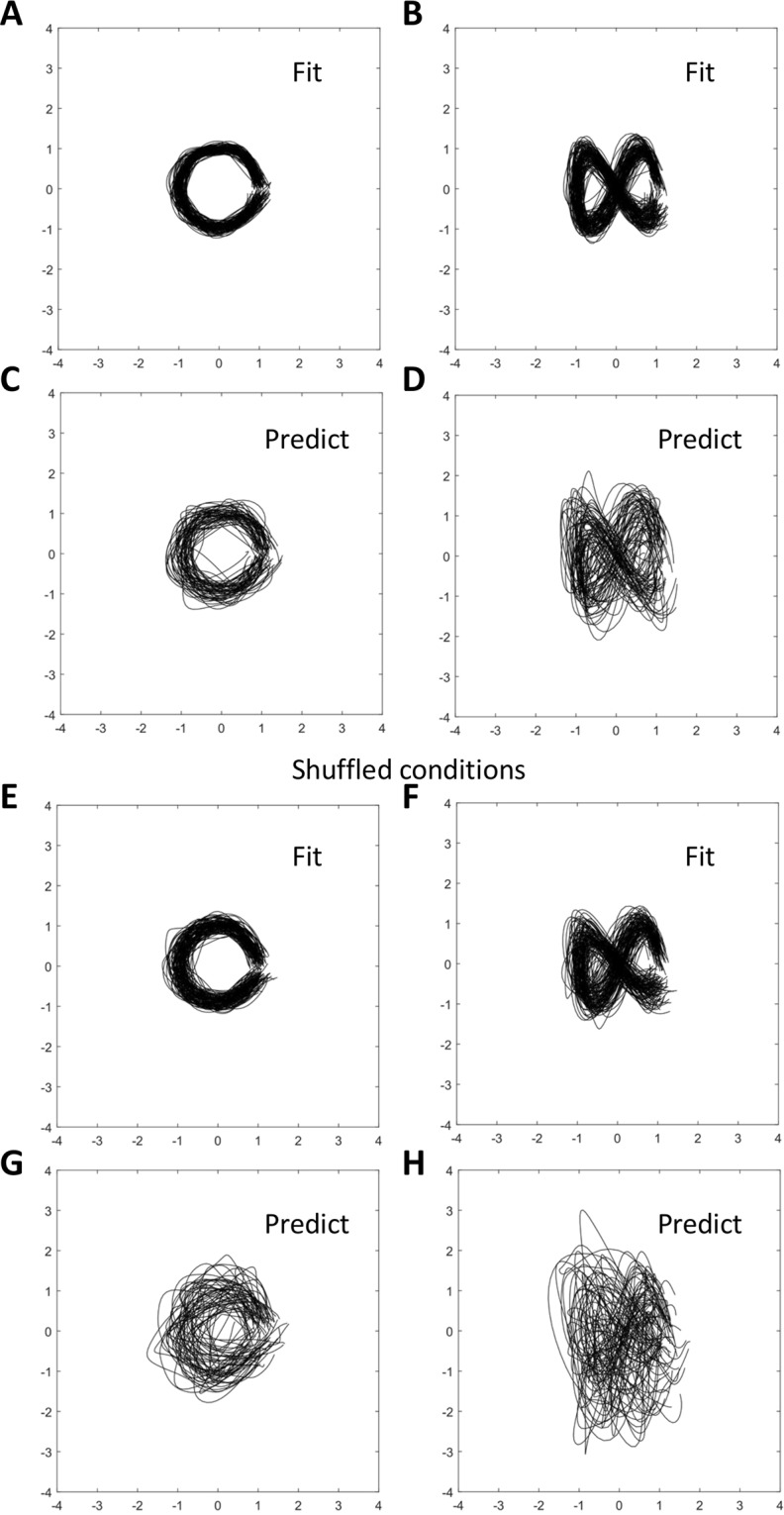
Fitting Churchland’s neuronal population data to Lissajous curves. (**A**) Fitting to a circle. (**B**) Fitting to the curve shaped as ∞. (**C**) Prediction of a circle. Half of the conditions were used to train the regression model; the other half to generate prediction. (**D**) Prediction of the ∞ shape. (**E–H**) Fitting and predicting for the data with shuffled conditions.

Finally, we probed a more direct approach for converting Churchland’s data into circular trajectories. Churchland *et al*. linearly fit the population activity vector to its first derivative (Eq. 1) with the goal of extracting a rotational structure. We hypothesized that such an extraction of a desired “population property” could be achieved with a multiple linear regression. Accordingly, we utilized multiple linear regression to fit neuronal data to a circle:

2 $$x=\,\cos \,\frac{2\pi {\rm{t}}}{{\rm{T}}};y=\,\sin \,\frac{2\pi {\rm{t}}}{{\rm{T}}}$$

or to the Lissajous curve shaped as ∞:

3 $$x=\,\cos \,\frac{2\pi {\rm{t}}}{{\rm{T}}};y=\,\sin \,\frac{4\pi {\rm{t}}}{{\rm{T}}}$$

where *t* is time and *T* is trial duration. The regression worked well for both shapes (Fig. 9A,B), and it even generalized to new conditions, as evident from the analysis where half of the data were used for training the regression model and the other half for generating predictions (Fig. 9C,D). The shuffled data could be fit to the Lissajous curves, as well (Fig. 9E–H), although the prediction of ∞ was very noisy (Fig. 9H).

## Discussion

To clarify the neurophysiological significance of “rotational dynamics”, we conducted additional analyses of Churchland’s data and performed simple simulations. In the original dataset, we discovered a temporal order in which the neurons were activated and found that this order was relatively consistent across conditions. In our opinion, this is a useful observation that has not been described in sufficient detail in the original article of Churchland *et al*. and the publications that emerged from their study. We suggest that simple plots of population PETHs (e.g., Fig. 2) should be used to clarify any results on “rotational dynamics”. These plots could be very useful to clarify claims like the “rotational dynamics” persist even though motor-related patterns are different across condition^12^ or the recurrent neural network trained to generate arm-muscle EMGs develops rotational patterns^20^.

The existence of a temporal spread in neuronal peak rates (or response latencies) has been extensively documented in the literature. For example, waves of neuronal discharges similar to those shown in Fig. 2E,F have been depicted in numerous publications^21–26^. Sequential structure has been reported in the frequency domain, as well^27–29^. As to motor cortical patterns during reaching, Fig. 7 in Georgopoulos *et al*.^2^ shows a distribution of the times of onset of the first increase in discharge in motor cortical neurons for center-out arm reaching movements performed by a monkey. The onsets were calculated for the neurons’ preferred directions and ranged −300 to 500 ms relative to movement initiation time. In a more recent paper^30^, we have demonstrated such a spread for simultaneously recorded populations of neurons recorded in the motor and primary somatosensory cortical areas.

It may be true that no previous paper focused on the consistency of neuronal activation order for a range of movements. Yet, Churchland and his colleagues did not emphasize such consistency either and instead emphasized variability of individual neuronal responses across conditions. (“Responses were typically complex, multiphasic and heterogeneous”). According to their explanation, rotational patterns are principally a population phenomenon that persists despite individual neurons exhibiting different activity patterns for different arm reaches. The same interpretation of Churchland’s results can be found in more recent literature^17,31,32^. For example, according to Kaufman *et al*.^32^, “while individual neurons may exhibit confusing responses, the population-level, dynamical systems view may provide insight about how a circuit performs its key computations”. In other words, according to Churchland *et al*. and their followers, dimensionality reduction technique is needed for making sense of how neurons as a population modulate their activity as time passes.

Temporal sequences of neuronal responses that are consistent across a set of experimental conditions could occur for various reasons. For example, such a temporal order could be related to serial information processing by a neuronal circuit. Orderly response onsets have been reported for the activity of multiple cortical areas transforming sensory inputs into behavioral actions. For example, de Lafuente and Romo^33^ reported that a wave of neuronal activity travelled from the somatosensory cortex to the premotor cortical areas when monkeys performed a task that required perceptual judgment of a tactile stimulus. Sensorimotor transformation of this kind could be analyzed using a comparison of response characteristics for different cortical locations and different types of neurons. Unfortunately, jPCA makes such an analysis impossible because it lumps the activity of many neurons together (e.g. compare Fig. 2 with Fig. 4C).

Based on the results of our analysis, we question that Churchland’s results provide a strong evidence in favor of the dynamical-system that acts “like a spring box” that “could be released to act as an engine of movement” (an allegory used by Michaels, *et al*.^17^). This is simply because the mere presence of a temporal sequence of neuronal responses tells us very little about the properties of the system even if we assume that it is dynamical. Temporal lags between the responses of different neurons could occur for various reasons, including direct connectivity, common input, or association with different stages of information processing. jPCA would reveal rotations even in the combined data from different monkeys – an obviously artifactual cause for lags between the responses of different neurons, and there would not be a way to tell that this is not a “dynamical system”. (Unless correlations between the responses of different neurons are analyzed across behavioral trials; but such an analysis is not incorporated in the Churchland *et al*. approach).

The finding that neurons in some parts of the nervous system respond to a stimulus with different delays (and jPCA reveals rotations) is not surprising as it has been published in myriads of papers. For example, in sensory systems, peripheral receptors are activated first, followed by neuronal activity in the spinal cord, brainstem, thalamus and eventually cortex. Although this sensory processing chain in principle could be defined as a dynamical system, this definition alone does not illuminate any mechanism of sensory processing. By the same token, motor systems of the brain are well-known to perform sensorimotor transformations^34,35^. In Churchland’s case, this is the transformation of a go-cue and preceding instructions into a motor execution command. Saying that “rotational dynamics” occur during this sensorimotor transformation only adds terminology but does not clarify any concrete neuronal mechanism.

According to Churchland *et al*., “a focus on the dynamics that generate movement will help transcend the controversy over what single neurons in motor cortex code or represent”. While we agree with this general idea, we do not see how their jPCA results transcend any controversy, particularly given the fact that population rotations could be obtained with little effort using simple and quite arbitrary simulations that did not mimic any important property of motor cortical activity. Without a linkage to a specific function of the motor cortex, jPCA appears to be just a way to produce an illustration of the lags between the responses of different neurons.

Although analysis of response lags in different neurons could be valuable for understanding neural processing pipelines, we are not convinced that jPCA is the best tool for such an analysis. We observed cases where temporal sequences of responses were revealed with several methods but not with jPCA set to default, where for each neuron an across-condition response was subtracted from this neuron’s PETHs. Thus, shuffling Churchland’s data across conditions did not destroy the rotations revealed with non-jPCA approaches (Figs. 3B,D and 4B) but jPCA failed to produce circular trajectories in this case (Fig. 4D). This happened because jPCA was designed to reveal “those aspects of the neural response that differ across conditions”, to avoid “more trivial possibilities”, and to “interpret projections where activity unfolds differently across conditions”. These goals that are not entirely clear to us were achieved by calculating an across-condition average PETH for each neuron and then subtracting this average response from all PETHs for that neuron. When we removed this data preprocessing step from jPCA, jPCA generated circles for the shuffled data (Fig. 8B) and simulated temporal sequences of neuronal responses (Fig. 8C). Looking closer at the preprocessing procedure employed by Churchland *et al*., we find it arbitrary and questionable. The first concern is that this data manipulation violates the assumptions of Eq. 1, where true neuronal rates are used instead of neuronal rates corrected by subtracting an average contribution from different conditions. If Churchland *et al*. wanted to hypothesize that an average response influenced population dynamics, they should have incorporated this function of time in Eq. 1 and explained what brain structure could generate it. Unless such an explanation is provided, it is unclear why one would need to subtract an across-condition average from the neuronal response. Consider an example of a mass on a spring, a harmonic oscillator that could be described as a dynamical system^36^. It would make little sense to analyze the properties of this system by subtracting average responses for different conditions like different values of mass and spring constant, which influence the period of oscillations. (This example is relevant to motor control where biomechanical parameters are different for different movements). Calculation of an across-condition average is an artificial procedure because it requires aligning neuronal data on an event of interest, for example go-cue onset, movement onset, or Churchland’s “neuronal movement onset”. For data points distant from this aligning event, variability in neuronal responses would grow^37^, making the average PETH a less reliable description of neuronal activity as a function of time and, consequently, an ineffective factor for Churchland’s data correction. Additionally, this correction cannot be applied to continuous behaviors like target pursuit task^38^, where phase differences between the activity of different neurons are very likely to occur but the task is not composed of epochs corresponding to a discrete set of conditions. Churchland and his colleagues justified their correction procedure by the desire to remove “rotations that are similar for all conditions” because they prevent one “to gain multiple views of the underlying process, making it difficult to infer whether rotations are due to dynamics or to more trivial possibilities”. While they did not clarify the difference between the “dynamics” and “more trivial possibilities”, the consequences of the implemented data manipulation are not trivial. It may have caused aliasing, such as significant distortion of neuronal responses. Overall, this manipulation appears poorly justified and its benefits are unclear.

Mathematically, Churchland’s method to produce a rotating pattern from neuronal PETHs can be described as a linear transformation with a certain number of free parameters. With a sufficient number of free parameters, multiple linear regression can yield practically any desired curve^39^. For example, when we applied a multiple linear regression to Churchland’s data, we easily produced a variety of Lissajous curves. Because of the similarity of neuronal responses across conditions, this transformation even generalized from the training dataset (half of the conditions) to new data in the test dataset (the other half) (Fig. 8). Evidently, no one would claim that any of these arbitrarily chosen curves represents a physiologically meaningful neural population mechanism, although we used a linear transformation very similar to the one Churchland and his colleagues employed to generate their curves.

Altogether, our results challenge the conclusions of Churchland and his colleagues. Clearly, their effects simply reflect what one should expect by applying a linear transformation, such as PCA, to reduce the dimensionality of large-dimension data and then adjusting the resulting components to a new basis that reproduces a desired behavior (in this case rotation). PCA^10^ and independent component analysis^11^ were introduced twenty years ago by our group to extract correlated neuronal patterns and reduce dimensionality of neuronal-ensemble data (recently reviewed^31,40^). While the findings of Churchland and his colleagues can be viewed as an extension of this approach and a method to produce a visually appealing phase plot, we doubt that they have discovered any new neurophysiological mechanism, as claimed in their article. Indeed, the mere fact that neurons in a population respond at different times with respect to a go-cue tells very little about the function of these responses and does not discard any of the previously proposed representational interpretations, such as the suggestion that neuronal populations perform a sensorimotor transformation^34,35,41^, handle specific motor parameters^2–5^, and/or respond to movement-related sensory inputs^42–45^.

Cortical activity is dynamical in the sense that neuronal rates change in time. Differential equations^46^ and other mathematical methods^13,47–49^ are certainly applicable to modeling these dynamics. However, a much more thorough analysis compared to the method proposed by Churchland *et al*. is needed for making a major advance in our understanding of cortical mechanisms of motor control. In light of the exponential increase in simultaneous neural recording^7,50^ and also the future impact of new technologies such as NeuroPixels^51^, it is therefore perhaps an appropriate moment to emphasize that pitfalls may await if we rely too heavily on low-dimensional representations of population activity, such as jPCA.

## Methods

The data (single units and good multiunits recorded in monkey N; the data used to construct Fig. 3f of Churchland *et al*.) and MATLAB scripts were obtained from the Churchland lab’s online depository (https://www.dropbox.com/sh/2q3m5fqfscwf95j/AAC3WV90hHdBgz0Np4RAKJpYa?dl=0). The dataset contained PETHs for each neuron and each condition. We used the following commands to run Churchland’s code:

$$times=-\,{50}:{10}:{550};$$

$$jPCA\_params.numPCs={6};$$

4 $$[{Projection},\,Summary]=jPCA(Data,\,times,\,jPCA\_params);$$

This corresponds to the time range −50 to 550 ms and six PCs entered in jPCA.

To produce the plots shown in Fig. 2A–D, we stacked Churchland’s PETHs together, and Fig. 2E shows PETHs averaged across conditions. The average PETHs were used to find peak firing rates and the time of their occurrences. PETHs of Fig. 2A–E were sorted according to the sequence of these peaks of the average PETHs. To improve the display of phase shifts between the neurons, PETHs of Fig. 2A–E were normalized by subtracting the mean and dividing by the peak PETH value. This normalization was used only for plotting the graphs of Fig. 2A–E but not for calculating average PETHs for individual neurons (Fig. 2E) or neuronal subpopulations (Fig. 3).

In the PCA analysis (Fig. 4), we standardized PETHs for each neuron by subtracting the overall mean (i.e., average for all PETHs appended to each other) and dividing by the overall standard deviation (again, for all PETHs appended).

Simulated PETHs (Fig. 5) were computed in MATLAB as:

5 $$PETH=exp(\,-(times-tau){.}^{\wedge }2/50)\ast (0.2+rand(1));$$

where the time shift, *tau*, was selected to produce 10-ms increments of the delay for the neurons in the sequence (Fig. 6). Neither the width of the response not the amplitude (uniformly distributed from 0.2 to 1.2 in Eq. 5) were critical for the rotations to occur. However, to cope with the ill-conditioning of the population responses highly correlated across conditions, it was important to introduce temporal variability to the simulated PETHs. This was done by offsetting *tau* for all neurons by the same amount of time for several groups of conditions (Fig. 6C,D).

Multiple linear regressions (Fig. 9) were implemented in MATLAB (*regress* function). Here, neuronal activity was transformed into Lissajous curves. Fitting (Fig. 9A,B,E,F) was conducted by using the same conditions as the training and test data. Predictions (Fig. 9C,D,G,H) were computed by using half of the trials to train the regression model and the other half to test.

## Data Availability

Data and scripts are available at https://www.dropbox.com/sh/svex4f947uxo53x/AABkOnIS_ertXcp1xZ-U6gO-a?dl=0.

## References

[1] Evarts EV (1972). Activity of motor cortex neurons in association with learned movement. Int J Neurosci.

[2] Georgopoulos AP, Kalaska JF, Caminiti R, Massey JT (1982). On the relations between the direction of two-dimensional arm movements and cell discharge in primate motor cortex. J Neurosci.

[3] Kakei S, Hoffman DS, Strick PL (1999). Muscle and movement representations in the primary motor cortex. Science.

[4] Georgopoulos AP, Ashe J, Smyrnis N, Taira M (1992). The motor cortex and the coding of force. Science.

[5] Zhuang KZ, Lebedev MA, Nicolelis MA (2014). Joint cross-correlation analysis reveals complex, time-dependent functional relationship between cortical neurons and arm electromyograms. J Neurophysiol.

[6] Nicolelis MA (2003). Chronic, multisite, multielectrode recordings in macaque monkeys. Proc Natl Acad Sci USA.

[7] Schwarz DA (2014). Chronic, wireless recordings of large-scale brain activity in freely moving rhesus monkeys. Nat Methods.

[8] Averbeck BB, Lee D (2004). Coding and transmission of information by neural ensembles. Trends Neurosci.

[9] Nicolelis MA, Lebedev MA (2009). Principles of neural ensemble physiology underlying the operation of brain–machine interfaces. Nat Rev Neurosci.

[10] Chapin JK, Nicolelis MA (1999). Principal component analysis of neuronal ensemble activity reveals multidimensional somatosensory representations. J Neurosci Methods.

[11] Laubach M, Shuler M, Nicolelis MA (1999). Independent component analyses for quantifying neuronal ensemble interactions. J Neurosci Methods.

[12] Churchland MM (2012). Neural population dynamics during reaching. Nature.

[13] Pandarinath C (2018). Inferring single-trial neural population dynamics using sequential auto-encoders. Nat Methods.

[14] Garfinkel, A., Shevtsov, J. & Guo, Y. Modeling life: the mathematics of biological systems. (Springer, 2017).

[15] Lara AH, Cunningham JP, Churchland MM (2018). Different population dynamics in the supplementary motor area and motor cortex during reaching. Nat Commun.

[16] Golub, G. H. & Van Loan, C. F. *Matrix computations*. Vol. 3 (JHU press, (2012).

[17] Michaels JA, Dann B, Scherberger H (2016). Neural population dynamics during reaching are better explained by a dynamical system than representational tuning. PLOS Comput Biol.

[18] Hall TM, de Carvalho F, Jackson A (2014). A common structure underlies low-frequency cortical dynamics in movement, sleep, and sedation. Neuron.

[19] Xu W, de Carvalho F, Jackson A (2019). Sequential neural activity in primary motor cortex during sleep. J Neurosci.

[20] Sussillo D, Churchland MM, Kaufman MT, Shenoy KV (2015). A neural network that finds a naturalistic solution for the production of muscle activity. Nat Neurosci.

[21] Peyrache, A., Benchenane, K., Khamassi, M., Wiener, S. & Battaglia, F. Sequential reinstatement of neocortical activity during slow oscillations depends on cells’ global activity. *Front Syst Neurosci* **3**, 10.3389/neuro.06.018.2009 (2010). 10.3389/neuro.06.018.2009 PMC2805426 20130754

[22] Luczak A, Barthó P, Marguet SL, Buzsáki G, Harris KD (2007). Sequential structure of neocortical spontaneous activity *in vivo*. Proc Natl Acad Sci USA.

[23] Bulkin DA, Law LM, Smith DM (2016). Placing memories in context: Hippocampal representations promote retrieval of appropriate memories. Hippocampus.

[24] Kvitsiani D (2013). Distinct behavioural and network correlates of two interneuron types in prefrontal cortex. Nature.

[25] Gage GJ, Stoetzner CR, Wiltschko AB, Berke JD (2010). Selective activation of striatal fast-spiking interneurons during choice execution. Neuron.

[26] Ermentrout GB, Kleinfeld D (2001). Traveling electrical waves in cortex: insights from phase dynamics and speculation on a computational role. Neuron.

[27] Prechtl J, Cohen L, Pesaran B, Mitra P, Kleinfeld D (1997). Visual stimuli induce waves of electrical activity in turtle cortex. Proc Natl Acad Sci USA.

[28] Wang X-J (2010). Neurophysiological and computational principles of cortical rhythms in cognition. Physiol Rev.

[29] Rubino D, Robbins KA, Hatsopoulos NG (2006). Propagating waves mediate information transfer in the motor cortex. Nat Neurosci.

[30] Ifft P, Lebedev M, Nicolelis MA (2011). Cortical correlates of Fitts’ law. Front Integr Neurosci.

[31] Cunningham JP, Byron MY (2014). Dimensionality reduction for large-scale neural recordings. Nat Neurosci.

[32] Kaufman MT, Churchland MM, Ryu SI, Shenoy KV (2014). Cortical activity in the null space: permitting preparation without movement. Nat Neurosci.

[33] de Lafuente V, Romo R (2006). Neural correlate of subjective sensory experience gradually builds up across cortical areas. Proc Natl Acad Sci USA.

[34] Kalaska J (1991). Reaching movements to visual targets: neuronal representations of sensori-motor transformations. Seminars in Neuroscience.

[35] Georgopoulos AP, Lurito JT, Petrides M, Schwartz AB, Massey JT (1989). Mental rotation of the neuronal population vector. Science.

[36] Hirsch, M. W., Smale, S. & Devaney, R. L. *Differential equations, dynamical systems, and an introduction to chaos*. (Academic Press, (2012).

[37] Renoult L, Roux S, Riehle A (2006). Time is a rubberband: neuronal activity in monkey motor cortex in relation to time estimation. Eur J Neurosci.

[38] Li Z (2009). Unscented Kalman filter for brain-machine interfaces. PLOS One.

[39] Babyak MA (2004). What you see may not be what you get: a brief, nontechnical introduction to overfitting in regression-type models. Psychosom Med.

[40] Paninski L, Cunningham JP (2018). Neural data science: accelerating the experiment-analysis-theory cycle in large-scale neuroscience. Curr Opin Neurobiol.

[41] Kakei S, Hoffman DS, Strick PL (2003). Sensorimotor transformations in cortical motor areas. Neurosci Res.

[42] Bötzel K, Ecker C, Schulze S (1997). Topography and dipole analysis of reafferent electrical brain activity following the Bereitschaftspotential. Exp Brain Res.

[43] Alary F (1998). Event-related potentials elicited by passive movements in humans: characterization, source analysis, and comparison to fMRI. Neuroimage.

[44] Goldring S, Ratcheson R (1972). Human motor cortex: sensory input data from single neuron recordings. Science.

[45] Rosen I, Asanuma H (1972). Peripheral afferent inputs to the forelimb area of the monkey motor cortex: input-output relations. Exp Brain Res.

[46] Lukashin AV, Georgopoulos AP (1993). A dynamical neural network model for motor cortical activity during movement: population coding of movement trajectories. Biol Cybern.

[47] Moody SL, Zipser D (1998). A model of reaching dynamics in primary motor cortex. J Cogn Neurosci.

[48] Kawato M, Wolpert D (1998). Internal models for motor control. Sensory guidance of movement.

[49] Todorov E (2000). Direct cortical control of muscle activation in voluntary arm movements: a model. Nat Neurosci.

[50] Stevenson IH, Kording KP (2011). How advances in neural recording affect data analysis. Nat Neurosci.

[51] Steinmetz NA, Koch C, Harris KD, Carandini M (2018). Challenges and opportunities for large-scale electrophysiology with Neuropixels probes. Curr Opin Neurobiol.

